# The Inadequacy of Our Resources in Operative Dentistry*Written for the Forty-first Annual Meeting of the Mississippi Valley Dental Association.

**Published:** 1885-05

**Authors:** F. W. Sage


### The Inadequacy of Our Resources in Operative Dentistry.*

F. W. SAGE, D. D. S.

A few years ago some one invented this maxim for the guidance of operators, "a tooth that is worth filling at all is worth filling with gold." The saying became the sheet-anchor of many aspirants to high rank among operators. What difficulties had to be surmounted in order to preserve a reputation for unvarying consistency no one who has tried to discard all other filling materials needs to be told. If other evidence of the unsoundness of the assumption embodied in this maxim were wanting, it could be easily supplied by many silent witnesses in the shape of cement, gutta-percha, and other fillings inserted by first-class operators. Still the fact remains that desperate measures were for a long time and are still employed to render possible a universal application of the principle that gold is the only suitable material for filling teeth. Device after device was invented to enable the operator to master the difficulties of inaccessible cavities. A strong spirit of emulation among operators sprang up; extraordinary difficulties stimulated to extraordinary efforts to overcome them, and many ingenious appliances and methods were suggested and successfully practiced. As a natural result, a few men in the profession presently attained to lofty eminence, by reason of rare success in overcoming what to very many, were insurmountable obstacles to the use of gold foil. A new interest was lent to the discussions in our conventions and associations by the descriptions and demonstrations set forth in detail by these leaders of the profession. Was the adjustment of the coffer-dam in a difficult case a problem offered for solution, some exceptionally gifted operator was usually at hand to rise and explain how he proceeded in such cases. Was the question of retaining a clamp in position or of reaching a distant cavity broached, some genius was ready with his special appli-

*Written for the Forty-first Annual Meeting of the Mississippi Valley Dental Association.

ance of curious design to make the crooked paths straight. Practical demonstrations upon a patient at the clinic were not wanting, still some problem-solvers contented themselves with the use of the blackboard and a bit of chalk. Sometimes the use of an appliance to be constructed in accordance with the requirements of the special case was suggested. If upon actual trial, the construction of the suggested appliance was found to be too difficult for the average operator, or if having been constructed it was found to be inadequate to the end sought to be attained, what of that? The failure illustrated the inferiority of the poor inquirer's practical talent, and redounded all the more to the glory and honor of him who offered the suggestion. A remarkable state of affairs had come about and prevails to this day; to wit: we find a large body of professional men led on and encouraged by a very small minority of proficients in the use of a filling material which it would seem that few can hope to be able to use successfully. A remarkable state of affairs, truly, that the success of the few should serve chiefly to illustrate the hopeless incompetency of the many! Who is to blame for this state of affairs? Who is to be held responsible for the failure of honest efforts put forth to invent a filling material worthy to supplant gold foil? It would be idle to charge the individual members of the profession with a lack of interest in devising some improvement in this respect. Are any like Demetrius of old fearful of endangering their craft? Is the five or ten dollars an hour that dentists receive for packing gold into cavities the real obstacle to the progress of invention? The advocates of a new departure failed to revolutionize existing methods for the all-sufficient reason that they offered no new improvements, no better alternative. They pointed out the shortcomings of the existing methods and to that extent they acheived a success, of a negative character, it is true. They directed attention to the fact that the best operators in gold were not accomplishing all they claimed in the way of saving teeth. It was the bugle's blast calling a halt, in order to inquire whether or not gold foil was to be accepted as the ideal filling material. The statement included in the much critized affirmation of the "New

Departure's" advocates that "in proportion as teeth need filling gold is the worst material used for filling cavities" would not be ignored or thrust aside. If these image-breakers had simply committed themselves to the opinion that gold foil does not fulfill all the requirements of an ideal filling material and had refrained from expressly encouraging the use of other materials already tried and found wanting, they would probably have won many a frank avowal of acquiescence from the very ones who resisted their conclusions.

Looking at the matter from every standpoint the conclusion seems to force itself upon us that the ideal filling material remains to be discovered. Consider for a moment some of the principles involved in the use of gold foil. Direct access to the cavity to be filled, must be obtained even at the expense of healthy, sound tooth-substance. That is a fundamental teaching as regards the preparation of the cavity for the reception of cohesive foil. In how many instances do we find the practice of this principle inadmissible, or being admissible, in how many cases is it highly objectionable? This principle, if literally adhered to, would result in the exposure of many a pulp. At best, free mortising down to the site of cavities, only partly accomplishes the purpose of gaining free access, in numerous instances which might be recalled. The necessity for such a course of procedure is certainly to be deplored. When cements are used the principle is largely modified, to the relief certainly of the patient, at least. We mold cement into cavities with comparatively thin, friable walls, which we would not dare to leave, but would freely cut away were we about to use gold. Twelve or fifteen years ago gold-workers pointed with pride and satisfaction to large contour operations glittering in front teeth. It was called artistic; and yet these operations lacked the very first requisite of perfection in art, which is to conceal art. The novelty of the thing carried us (and our patients too) away with enthusiasm. But the incongruity of a large mass of gold glittering in a front tooth forced itself upon attention after a while, and to-day, persons of cultivated tastes would gladly avoid any display of gold whatever in their teeth. The growing demand is for something closely resembling enamel. We hear with incredulity the avowal of our wealthy patient that he would rather have cement or gutta percha in his front teeth for appearance sake. We think him niggardly or lacking appreciation of the higher order of dental art, or we attribute his whim to a fear of the coffer dam and the polishing strip. Admit that neither cement nor gutta percha would meet our fastidious patient's requirements: the fact remains that he objects to the appearance of gold.

But, about the possibility of saving teeth with gold, let us now inquire. This ideal filling material requires an ideal tooth upon which to exhibit its saving efficacy. Gold is the veriest autocrat. It says to the operator, adapt your conditions to my requirements and I will fulfill your wishes, provided you give close enough heed to such requirements as I may choose to impose later. I pay no attention to the requirements of existing conditions. I dictate my terms. You meet them as best you may. I know that I am accounted stubborn and exacting. What care I for your opinion of me, good or bad? I have one or two peculiar properties distinctively my own. These properties are indispensable to your success in your calling. Attempts have been made to usurp my supremacy as the king of filling materials but all have failed. I have my faults, glaring faults, if you please, but still you dare not disown me.

So the profession goes on using gold, cutting grooves along the friable edges of incisors in order to find an attachment for gold. The end is accomplished; the glittering mass clings like a parasite to the sensitive surface, and the triumph of gold is complete, albeit the bordering enamel may crumble, lateral walls break away bodily and cervical borders soften. The tooth may be thick, dense of structure, tough and devoid of sensibility; or it may be thin and translucent, imperfectly calcified, brittle, and exquisitely sensitive; it is all one, we will adhere to the maxim and fill it with gold. It may not be the best conceivable filling material, but it is the best thus far discovered.

Perhaps it would not be utterly idle and preposterous to consider what we would like to have in the way of an ideal filling material. You royalists who shudder at the thought of gold's being dethroned, calm your fears, for the fulfillment of the prophecy belongs perhaps to the distant future. The ideal filling material should be a plastic, it should of course be indestructible, a non-conductor, compatible in every respect, color and appearance included, with tooth substance. It should possess the valuable quality peculiar to some of the cements now used, of attaching itself to and supporting comparatively frail walls of enamel. It should be easily and quickly introduced, and should be susceptible of a fine polish. In short it must combine the desirable qualities of gold and all other filling materials now in use, and in addition to these a few other qualities which the inventor will be likely to call to mind. How almost invaluable would be a material which would firmly adhere to a smooth surface of dentine without the aid of grooves or undercuts. How much mischief by the way, has been done by cutting retaining grooves; how many apparently strong walls of enamel have been undermined and checked? To be sure, we who fill with gold, or tin, or amalgam, have no alternative but to groove, if we would not have our fillings drop out. But the ideal filling material, which is expected to adhere to a smooth, flat surface, will obviate all that.

What a dismantling of our dental cabinets will result when the new ideal filling material actually displaces gold forever. Behold our fine sets of Varney's, and Butler's, and Chappell's, and Watling's pluggers forged over and made into excavators! Behold the decline of the finishing-bur, the corundum-point, the porte-polisher, the finishing-file, the polishing-strip, automatic, pneumatic, electric, and-so-forth-plugger, the file carrier, the polishing-buffs, Hindo-stan, Scotch, Arkansas and other wheels, the matrix, the burnisher, the foil-shears and crimpers, foil-carriers, the retaining-screw, the retaining-point drill, and finally, the stupendous yarn about the incredible consumption of gold by the $30,000 a year operator who never, not even hardly ever, uses amalgam. Behold the decline in the consumption of coffer-dam rubber and silk ligatures, and in short, behold the changed visage of the dental depot's clerk when the dentist orders another fifty cent package of cement and says never a word about being nearly out of gold, and felt-foil, and amalgam, and mercury, and Hill's stopping, and oxide of tin, etc.

In a decade or two our forceps will all have been remodelled into can-openers, and our elevators converted into tack-pullers, while the nitrous oxide gas artist will join a circus or turn veterinary surgeon. The dental engine, the spatula, the excavator and the chisel only, will survive in all their pristine glory and vigor. No longer will the controversy wax hot in the dental convention over the merits of No. 60 as compared with No. 4 foil, or over the respective merits of grooves and retaining-pits. The exposed pulp will probably still come in for a share of attention, and pyorrhea alveolaris will be met with a specific in the shape of an unrestricted opportunity to all the members to talk it to death.

What of the future? Fifty years hence another Barnum will scour the country for a man or woman with a Richmond crown. Five years hence—that is to say five years after the beginning of the looked-for New Era, dentists will charge a uniform price of one dollar per filling and they will fill ten teeth where they now fill one. Do not shrink so apprehensively from the inevitable, my dear professional brother. Your patrons are not going to forsake you to patronize some fifty cent man across the way. Skill and judgment and care have been your capital in the past and so they will be in the coming future. If you keep your hands and face clean, and don't reverse your collar to often, you may expect a much larger patronage then than you ever enjoyed in the past. Those who have given their years to the study of the teeth, their anatomy, their pathological conditions, their relations to other organs, have but little to fear from an influx of new-comers into the profession, for dental education has already reached such a stage of development that the public have become informed and are able to discriminate between true attainments and mere pretense.

Seriously, we ask, is the forecast of the coming event as we have sketched it, quite improbable? Who shall say, in this age of astounding invention, that a simple, durable, in every respect satisfactory substitute for gold foil filling will not readily be discovered? What if some one not in the profession should announce the discovery and should organize a joint stock company of shrewd business men to make known its genuine merit, could we, if we were so selfish and ungenerous as to wish it, prevent the revolution which the public would at once demand? Who believes such a thing as successful opposition would be possible?

Does the contemplation of the bare possibility of what we have suggested cause heart-burnings, and must we then confess that the abolition of gold foil would cause the carefully constructed fabric which we have been taught to believe was long ago firmly established on a corner stone of professionalism to tumble into ruins? Perish a thought so unworthy! Perish the pride that couples the word professional with the worship of the golden calf! Where is all our profession of concern for the public's welfare? Has it vanished with the reflection that our elaborate measure for the prevention and relief of human suffering may yet be supplanted by other means far more simple and more easily applicable? Did time and space permit, it would be interesting to trace out at length the results, both as concerned the dentist and the public, of such a material being introduced. The thousands upon thousands, who at this day never resort to a dentist, would become his patrons. For it is not merely the expense of operations which deters many from the attempt to save their teeth. Fear is a powerful deterrent. If it were the case that we could dispense with the painful clamp, the nerve-rasping groove-cutter, the retaining-point drill, the disagreeable corundum-tape and the annoying rubber-dam, would not our operations be robbed of more than half their terrors? Who has not observed that the simple removal of the decay in a cavity may be accomplished with comparatively little pain in a majority of instances? It is the undercutting and drilling to secure a retaining shape that hurts most. What an impetus would be given to the cause of dental education by this revolution. The dentist would then find more occasion than ever to extend his sphere of practical usefulness by studying the diseases of the nose, throat, and other contiguous parts. Indeed this idea has already gained a foothold among some of the young members of our profession in this city, and the medical colleges have opened their doors to more than one D.D.S. within the past five years. It is possible then that the destiny of the dentist, a few years hence, will be entirely changed. Is there any occasion for regret that this may happen, when it is more than probable that it would result in the opening to us of a broader and more liberal field? We think not. Be all this as it may, there is no questioning the fact that with the introduction of such a filling material as we have tried to describe, such an overturning and such an upheaval in the department of operative dentistry would occur as the wildest flight of imagination could hardly exaggerate. Will it come? If it comes are we prepared to show that gold foil is not the foundation stone of our professional pretensions?

When photography took its place among the arts, portrait painting declined. The artist remained an artist, but he underwent a metamarphosis. What will be our fate when the great improvement comes in?

